# Distinguishing Between Innocent and Pathologic Pediatric Heart Murmurs: A Case Report

**DOI:** 10.7759/cureus.111177

**Published:** 2026-06-19

**Authors:** Kelsey I Thomas

**Affiliations:** 1 Department of Medicine, University of California, Los Angeles, Los Angeles, USA

**Keywords:** atrial septal defect (asd), cardiac exam, heart murmur, pathologic murmur, pediatric heart murmur

## Abstract

Pediatric heart murmurs are common findings during physical examination, but only a small number of cases require intervention. This case report highlights a routine physical examination for a 10-year-old male patient that revealed a grade III/VI harsh systolic murmur heard in all areas of auscultation and a fixed split S2. A subsequent transthoracic echocardiogram showed an atrial septal defect that required catheter-based closure. This case proved to be one of the few cases in which urgent treatment of the heart murmur is required, and it helps highlight the features of pathologic murmurs that should prompt further evaluation.

## Introduction

Heart murmurs are common physical examination findings during routine well-child visits. It is critical for physicians who treat children to be able to distinguish between innocent murmurs (also frequently called flow, physiologic, or functional murmurs) and pathologic murmurs caused by turbulent flow through a congenital defect. Among older children, only 1% of those with a murmur have structural heart disease that requires intervention [1]. An atrial septal defect is a congenital defect most commonly detected in later life and constitutes 8%-10% of all congenital heart defects [2]. Though some children with large atrial septal defects may remain asymptomatic, it is important that large defects causing left-to-right shunting are closed to prevent long-term strain on the right side of the heart [3]. Without the ability to differentiate between innocent and pathologic murmurs, physicians may pursue unnecessary testing that creates additional stress and anxiety for the patient and their family [4], or may miss diagnosing a congenital heart defect that would have required intervention.

## Case presentation

A 10-year-old boy with no significant past medical history presented as a new patient for his annual well-child check. His family had no particular concerns, and he had no family history of congenital heart disease. His vital signs included a blood pressure of 96/63, a heart rate of 85, and an oxygen saturation of 98% on room air. His growth percentiles were within normal limits and appropriate for his prior growth trajectory. His physical examination was notable for a grade III/VI harsh systolic murmur heard across all areas of auscultation and a fixed split S2. There was no S3 or S4 present on auscultation, and no cyanosis or clubbing of his fingers.

Due to the unusually loud volume of the murmur, large area of involvement, and fixed split S2, an electrocardiogram was completed. The EKG showed a normal sinus rhythm and did not meet criteria for left ventricular hypertrophy or right ventricular hypertrophy. A transthoracic echocardiogram was also ordered to evaluate for any congenital defects.

**Figure 1:**
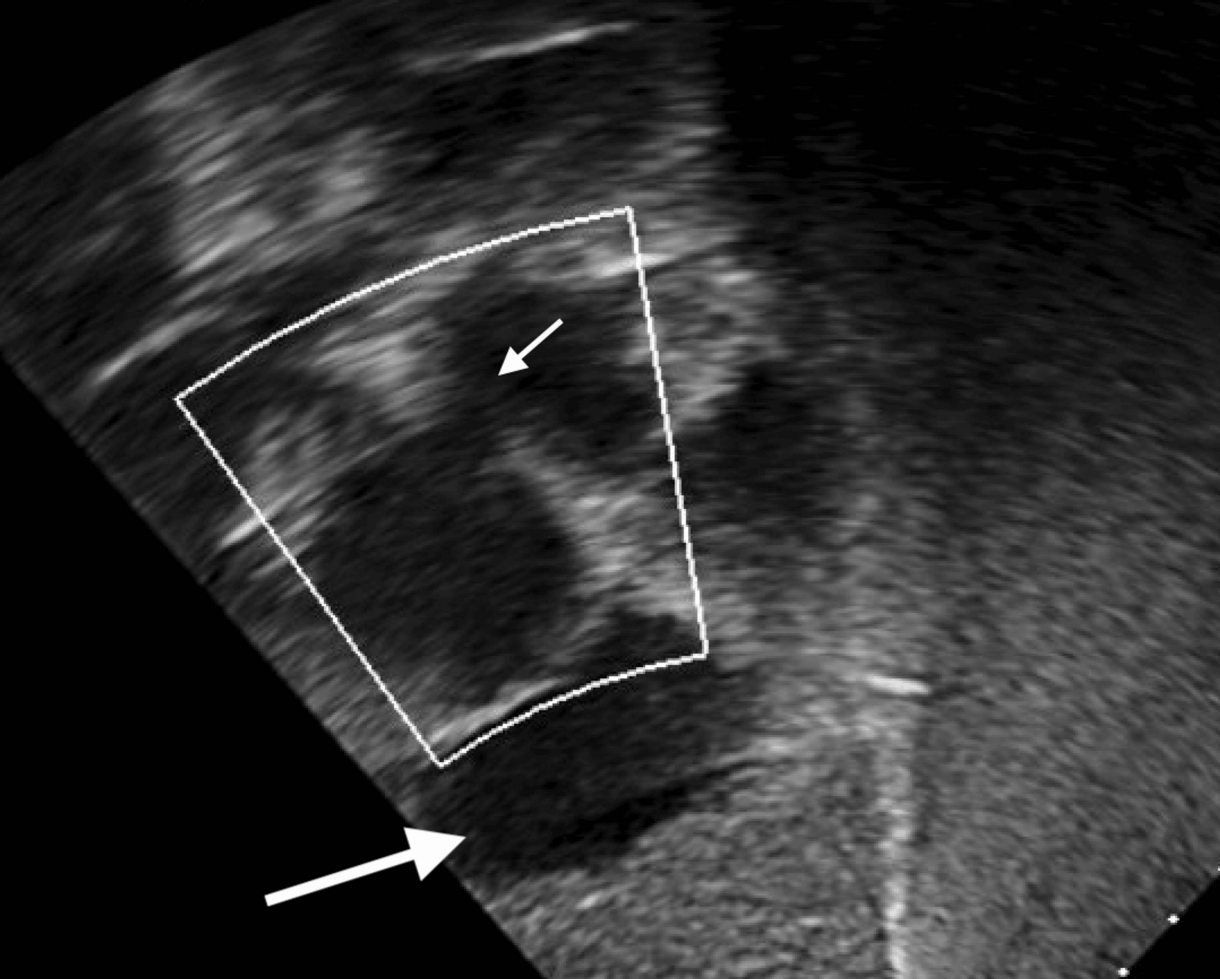
Transthoracic echocardiogram demonstrating a large secundum atrial septal defect between the left and right atria (small arrow) and right ventricular enlargement due to shunting of blood flow to the right side of the heart (large arrow).

The transthoracic echocardiogram demonstrated a large 2cm secundum atrial septal defect with all left-to-right flow, as well as a dilated right atrium, moderate to severe right ventricular enlargement, and dilated main and branch pulmonary arteries (Figure 1).

**Figure 2:**
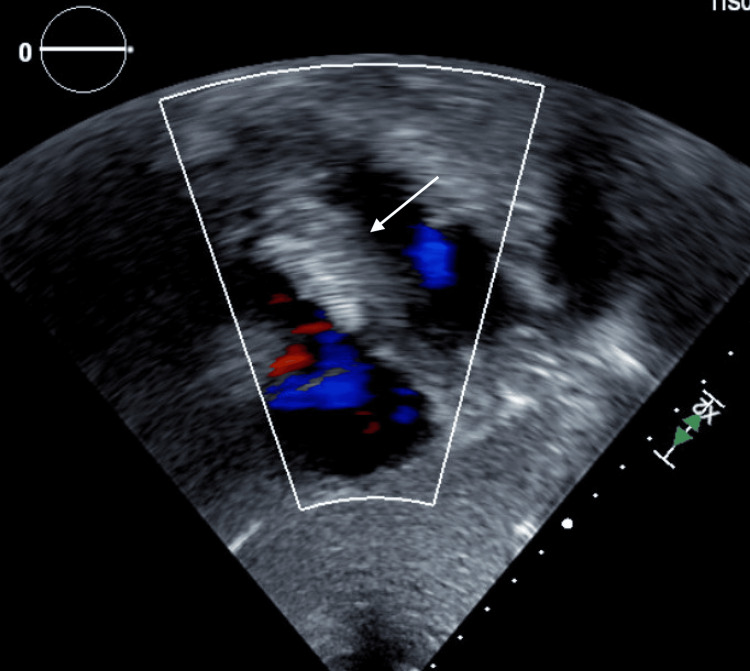
Transthoracic echocardiogram demonstrating the 26mm Amplatzer Septal Occluder positioned across the atrial septum with no residual shunting.

The patient was then seen urgently by pediatric cardiology on the same day as his echocardiogram. Within two weeks of diagnosis, he underwent catheter-based closure of a 26mm secundum atrial septal defect using an Amplatzer Septal Occluder (AGA Medical, Plymouth, Minnesota, USA). Post-procedure transthoracic echocardiogram showed no residual shunting and good biventricular function (Figure 2). His recovery after the procedure was uncomplicated.

## Discussion

Murmurs are a common physical examination finding among pediatric patients, with up to 8.6% of infants and 80% of young children diagnosed with a murmur at some point. Among infants with a murmur, 37% are later diagnosed with congenital heart disease, and 2.5% have defects that require procedural intervention [5]. Murmurs among older children are more often innocent, but it is still important to accurately identify murmurs that require further evaluation.

When auscultating a murmur, attention should be paid to the timing, duration, intensity, location, configuration, quality, pitch, and radiation of the murmur [2]. Innocent murmurs generally follow the Rule of the Seven S’s: soft, systolic, small area of involvement, short duration, single (without clicks or snaps), sweet (as opposed to harsh), and sensitive to position changes [6]. The most common innocent murmurs in older children are Still’s murmur, pulmonary flow murmur, and venous hum. Still’s murmur is a soft, low-frequency systolic murmur heard best between the left sternal border and apex of the heart. It is most commonly heard in children aged three to six years, and should disappear by the teenage years. Pulmonary flow murmurs are systolic murmurs heard best over the pulmonary valve (at the left upper sternal border) and do not radiate. They are the most common murmurs heard in adolescents and may be present between the ages of 5 and 14 years. Venous hum is a soft, continuous murmur heard best around the clavicles when the patient is sitting upright, typically between the ages of three and six years [2,6].

Conversely, signs of a pathologic murmur in an older child include any diastolic murmur, a loud or harsh-sounding murmur, a holosystolic murmur, a murmur that radiates to the back or neck, or any symptoms of heart disease. The presence of any extra heart sounds (fixed split S2, presence of S3 or S4, precordial thrills, or clicks) is also abnormal and can indicate a pathologic murmur [6]. In the case of our patient, a loud and harsh character to the murmur and a fixed split S2 raised suspicion that the murmur may be pathologic and required further evaluation.

Certain physical exam maneuvers can also help distinguish innocent from pathologic murmurs. The Valsalva maneuver decreases preload and increases the intensity of a murmur due to hypertrophic obstructive cardiomyopathy (HOCM) and mitral valve prolapse (MVP) [2,6]. Alternatively, having the patient squat or complete a passive leg raise increases preload and decreases the intensity of these murmurs. Hand grip increases afterload, which increases the intensity of murmurs due to mitral regurgitation, pulmonic stenosis, or ventricular septal defect, and decreases the intensity of murmurs due to HOCM, MVP, or aortic stenosis [6]. Notably, if a murmur disappears when a patient stands, this has a 98% positive predictive value for the murmur being innocent [7].

Though new pathologic murmurs in older children are uncommon, the congenital defects most often missed in early childhood are the bicuspid aortic valve and atrial septal defect [2]. Between the two, atrial septal defects are the defects most commonly detected later on, as was the case for our patient. His murmur was particularly harsh and loud, and it had a large area of involvement. The fixed split S2 was also characteristic of an atrial septal defect, as it was caused by delayed closure of the pulmonic valve resulting from increased filling of the right ventricle.

While echocardiography is the method by which cardiac defects are diagnosed, it is now recommended that patients with a possible pathologic murmur be referred to cardiology before ordering imaging, as this has been found to be more cost-effective [8]. In addition, routine EKG and chest x-ray are not recommended for the diagnosis of murmurs in the absence of other signs of dysrhythmia or pulmonary complaints. Based on this guidance, the EKG completed at our patient's visit was unnecessary as an initial screening test, and he could have been referred directly for pediatric cardiology consultation.

In patients diagnosed with an atrial septal defect, closure is indicated when there is significant left-to-right shunting, as evidenced by right heart enlargement due to volume overload. Even if the patient is asymptomatic, closure is recommended in these cases to reduce further volume overload. Closure may also be considered for small defects with no right ventricular volume overload if there is evidence of right-to-left shunting causing hypoxemia or paradoxical embolism [3]. In the case of our patient, the atrial septal defect was large enough to cause significant shunting that led to right atrial dilation and right ventricular enlargement, necessitating closure.

## Conclusions

Most heart murmurs heard in older children are innocent, but some congenital defects can be missed in early childhood or may only produce a pathologic murmur later on in life. It is important for physicians to distinguish between innocent and pathologic murmurs during an examination so that patients get prompt and appropriate referrals. In general, innocent murmurs are soft, systolic, short, small, single, sweet, and sensitive to position changes. Innocent murmurs should also disappear with standing and decrease with the Valsalva maneuver. Murmurs that do not have these characteristics should ideally prompt a referral to cardiology or, if specialist availability is limited, an echocardiogram to establish the diagnosis.
